# Overexpression of *TcCHS* Increases Pyrethrin Content When Using a Genotype-Independent Transformation System in Pyrethrum (*Tanacetum cinerariifolium*)

**DOI:** 10.3390/plants11121575

**Published:** 2022-06-15

**Authors:** Jiawen Li, Zhizhuo Xu, Tuo Zeng, Li Zhou, Jinjin Li, Hao Hu, Jing Luo, Caiyun Wang

**Affiliations:** 1 Key Laboratory for Biology of Horticultural Plants, Ministry of Education, College of Horticulture & Forestry Sciences, Huazhong Agricultural University, Wuhan 430070, China; lijiawen@webmail.hzau.edu.cn (J.L.); xzz1122@webmail.hzau.edu.cn (Z.X.); zengtuo@gznu.edu.cn (T.Z.); zhouli@webmail.hzau.edu.cn (L.Z.); jinjinsweet@mail.hzau.edu.cn (J.L.); haohu@mail.hzau.edu.cn (H.H.); 2 School of Life Sciences, Guizhou Normal University, Guiyang 550025, China

**Keywords:** *Tanacetum cinerariifolium*, pyrethrins, transformation, shoot apical meristem, regeneration recalcitrance

## Abstract

Pyrethrum (*Tanacetum cinerariifolium*) is one of the most important industrial crops for the extraction of pyrethrins, which are natural insecticidal compounds. Progress in pyrethrum molecular breeding with the objective of increasing pyrethrin content has been slow for lack of a suitable gene transfer system. Regeneration recalcitrance is a crucial barrier to establishing a genetic transformation system in pyrethrum. Therefore, in this study, an Agrobacterium-mediated transformation system in pyrethrum was developed using shoot apical meristems from germinated seedlings. Factors affecting transformation efficiency were optimized. Optimal conditions included explants at the “no true leaf” stage with a half apical meristem, an *Agrobacterium tumefaciens* cell density of OD_600_ = 0.5, two days of cocultivation, and the incorporation of 1.5 mg L^−1^ 6-BA and 30 mg L^−1^ kanamycin into the selection medium. Under the optimized conditions, two expression cassettes (*proTcCHS-GUS* and *proRbcS-TcCHS*) were successfully transformed into pyrethrum. Polymerase chain reaction (PCR), Southern blotting, reverse-transcription quantitative PCR (RT-qPCR), and histochemical staining confirmed the identity of *proTcCHS-GUS* transgenic plants. PCR and RT-qPCR analyses confirmed the identity of *proRbcS-TcCHS* transgenic plants. The transformation efficiency was 0.83% (5 transgenic lines/600 infected explants). The relative concentration of pyrethrins in *proRbcS-TcCHS* transformants (OX T0-1: 1.50% or OX T0-2: 1.24%) was higher than that in nontransformed plants (WT: 0.76%). Thus, the genetic transformation system overcame the low regeneration efficiency and integrated a foreign gene into the pyrethrum genome. The new system is a suitable and effective tool for creating high-yielding cultivars of pyrethrum.

## 1. Introduction

Pyrethrum (*Tanacetum cinerariifolium*) is a perennial herbaceous plant that has been cultivated for centuries as a source of pyrethrins [1], which are natural insecticidal compounds, including pyrethrin I, cinerin I, jasmolin I, pyrethrin II, cinerin II, and jasmolin II [2]. These compounds are highly toxic to a variety of insects, but are nontoxic to mammals [3]. Given their favorable qualities, there is tremendous global demand for pyrethrins in order to avoid the overuse of synthetic alternatives in crop production [4]. Many studies have investigated the mechanism of pyrethrin biosynthesis [5,6,7,8]. However, the molecular breeding of pyrethrum has been hampered due to a lack of a suitable genetic transformation systems. The primary obstacle to this is that pyrethrum genotypes are resistant to regeneration after genetic manipulation [9].

The infection of explants with *Agrobacterium tumefaciens* followed by regeneration from transgenic cells has been successfully used for the genetic transformation of many plant species. However, there is only one report of *A. tumefaciens*-mediated genetic transformation in pyrethrum (clone No. 39), and the approach requires a unique radiation-induced mutant [10]. However, this method has not been replicated in subsequent studies. The reason for this may be that the mutation caused by radiation treatment is unstable [11]. In addition, elite commercial pyrethrum may lack a strong regeneration capacity. Therefore, the development of a transformation method that is independent of specific genotypes is required.

Shoot apical meristem explants have axillary meristems present at the junction of the cotyledon and embryo axis [12]. These meristematic cells can develop into shoots without redifferentiation or dedifferentiation [13]. Thus, genetic transformation using apical meristems as the target material is considered to be genotype-independent [14]. Shoot apical meristems have been used to generate transgenic plants in many transformation systems, including in the gene gun bombardment of *Gossypium hirsutum* [15], *Ricinus communis* [16], and *Zea mays* [17], and the Agrobacterium-mediated transformation of *Lupinus angustifolius* [12], *Corchorus capsularis* [18], *Cicer arietinum* [19], *Oryza sativa* [20], *Cucumis sativus* [21], and *Eleusine coracana* [22], to overcome regeneration recalcitrance in genetic transformation.

In this study, a new method for Agrobacterium-mediated genetic transformation in pyrethrum is proposed that takes advantage of the inherent properties of shoot apical meristems. Using this system, the *TcCHS* gene (chrysanthemol synthase, CHS), which is a crucial enzyme in pyrethrin biosynthesis, is overexpressed in transgenic pyrethrum. This novel method will expedite future research and the molecular breeding of pyrethrum, and potentially other dicotyledons.

## 2. Results

### 2.1. Optimization of Factors Affecting Shoot Induction and Transformation

**Figure 1:**
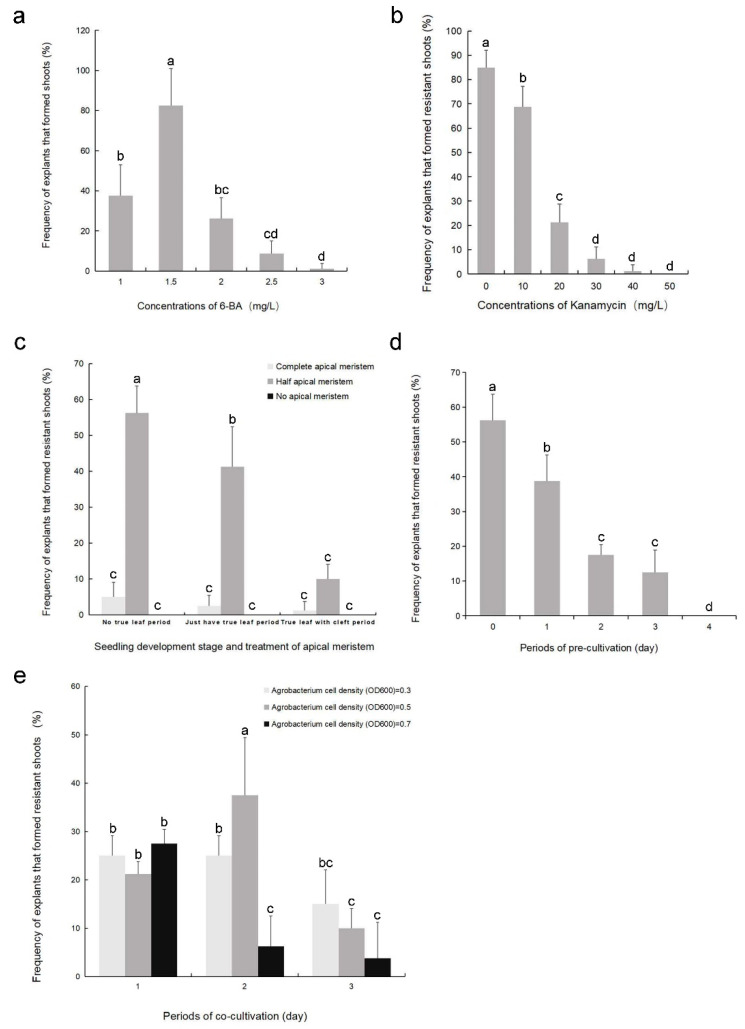
Factors affecting the efficiency of pyrethrum shoot induction and transformation. Effects of (**a**) 6-benzylaminopurine (6-BA) concentration on shoot induction, (**b**) kanamycin concentration on shoot induction, (**c**) explant status on transformation, (**d**) preculture duration on transformation, and (**e**) cocultivation period and the concentration of *A. tumefaciens* on transformation. Bars indicate the mean ± SD. Bars with a different lower-case letter are significantly different (LSD test, *p* < 0.05).

The effects of 6-benzylaminopurine (6-BA) and kanamycin concentrations were evaluated based on the frequency of explants that formed shoots. The highest frequency (82.5%) was obtained with 6-BA at 1.5 mg L^−1^ (Figure 1a). Therefore, 1.5 mg L^−1^ 6-BA was selected for shoot induction. After 14 d of exposure to 30 mg L^−1^ kanamycin, the explants gradually whitened and almost all shoots stopped growing (Figure 1b). Therefore, 30 mg L^−1^ kanamycin was selected to supplement the selection medium in all subsequent transformation experiments.

Next, *A. tumefaciens* harboring the pBI121 vector (*pro35S-GUS*) were used in transformations, and the effects of various experimental factors on the transformation efficiency of pyrethrum were evaluated based on the frequency of explants that formed kanamycin-resistant shoots. Transformation efficiency declined significantly with seedling age (ranging from no true leaf to a true leaf with a cleft), and almost all kanamycin-resistant shoots developed from explants containing half apical meristems (i.e., cut longitudinally into two halves). The highest transformation efficiency (56.3%) was obtained with explants at the “no true leaf” stage with half apical meristems (Figure 1c). The preculture duration of the explants also significantly affected transformation efficiency. Transformation efficiency decreased significantly with prolonged preculture, and nonprecultured explants showed the highest transformation efficiency (56.3%; Figure 1d). The optimal duration of cocultivation and optimal *A. tumefaciens* concentration were assessed; kanamycin-resistant shoots were induced in all tested conditions. However, on day 14, some experimental groups had serious *A. tumefaciens* contamination, and some shoots were necrotic. Cocultivation for 2 d with an *A. tumefaciens* optical density of 600 nm (OD_600_) at 0.5 resulted in the highest transformation efficiency (37.5%; Figure 1e), and thus was considered the optimal setting.

### 2.2. Genetic Transformation and Selection of Putative Transgenic Pyrethrum Plants

**Figure 2:**
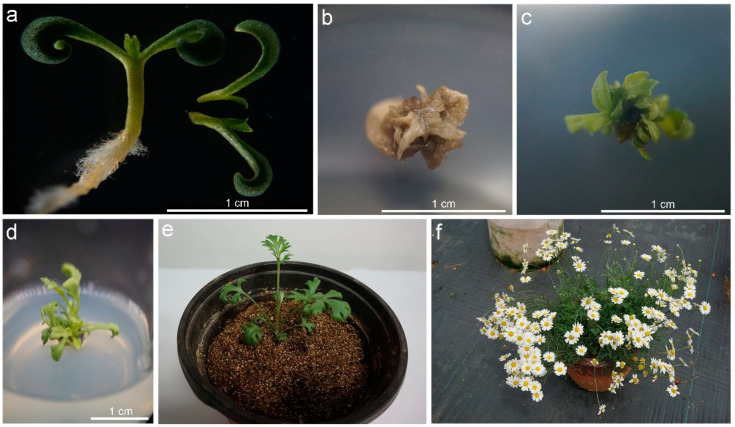
Induction of transgenic *proTcCHS-GUS* plants from pyrethrum shoot apical meristems. (**a**) Seedlings of pyrethrum were germinated in sterile conditions and seedling shoot tips were cut in half longitudinally. (**b**) After 4 weeks of selective culture, nontransformed shoots turned brown. (**c**) After 4 weeks of selective culture, transformed explants formed kanamycin-resistant shoots. (**d**) Kanamycin-resistant shoots were transferred to root-induction medium for 1 month. (**e**) Rooted shoots were transferred to sterile vermiculite for 30 d. (**f**) Adapted plants were transferred to soil for 1 year. Scale bar = 1.0 cm.

The successful Agrobacterium-mediated transformation of pyrethrum apical meristems was achieved based on the optimized conditions. *A. tumefaciens* (EHA105) harboring the *proTcCHS-GUS* vector was used in transformations. Germinated seedlings were used as experimental material (Figure 2a). Following transformation, explants were transferred to shoot-induction medium supplemented with 30 mg L^−1^ kanamycin. Nontransformed shoots turned brown within 4 weeks (Figure 2b). Some transformed explants formed kanamycin-resistant shoots after 4 weeks of culture (Figure 2c). Kanamycin-resistant shoots were then transferred to root-induction medium (Figure 2d). Rooted shoots were propagated and transferred to sterile vermiculite for 30 d (Figure 2e). Adapted plants were transferred to soil (Figure 2f).

### 2.3. Verification of Transgenic proTcCHS-GUS Plants

**Figure 3:**
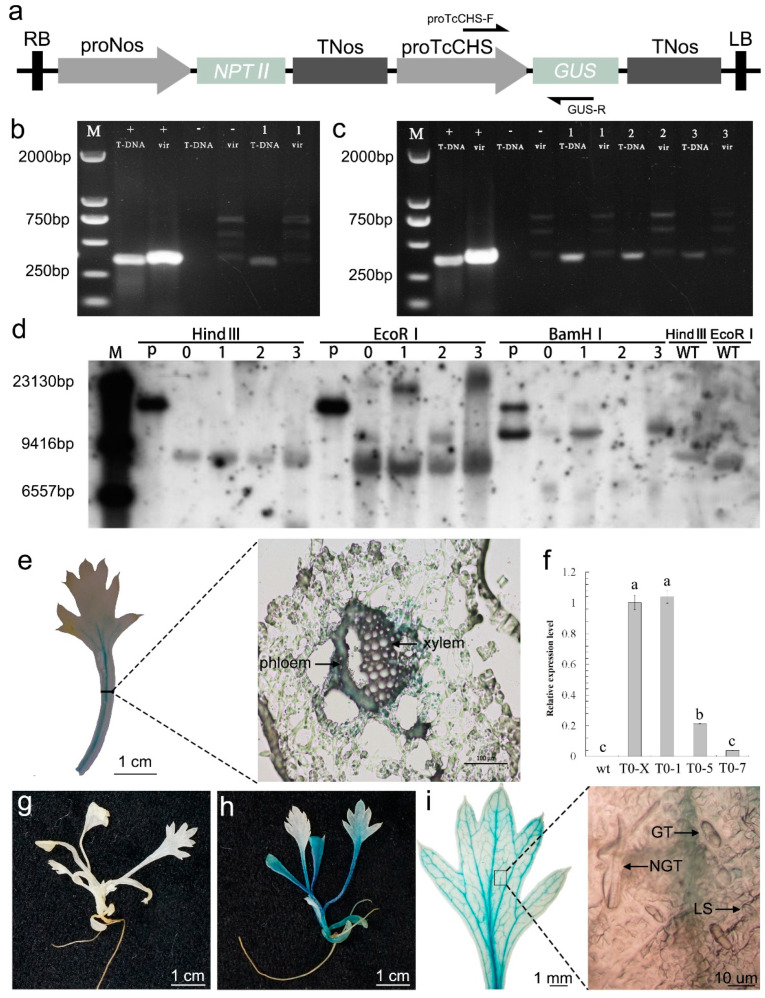
Molecular analysis of transgenic *proTcCHS-GUS* pyrethrum plants. (**a**) Schematic structure of the *proTcCHS-GUS* vector and primer locations (arrows). (**b**) Agarose gel image of PCR products showing amplification of the *proTcCHS-GUS* and *VirG* gene from genomic DNA of T0-X. M: marker; +: EHA105 harboring *proTcCHS-GUS* vector; −: control from nontransformed plants; 1: T0-X; T-DNA: proTcCHS-F and GUS-R were used for PCR; *vir*: VirG-F and VirG-R were used for PCR. (**c**) Agarose gel image of PCR products showing amplification of the *proTcCHS-GUS* gene and *VirG* gene from genomic DNA of T0-1, T0-5, and T0-7. M: marker; +: EHA105 harboring *proTcCHS-GUS* vector; −: control from nontransformed plants; 1: T0-1; 2: T0-5; 3: T0-7; T-DNA: proTcCHS-F and GUS-R were used for PCR; *vir*: VirG-F and VirG-R were used for PCR. (**d**) Southern blot of T0-X, T1-1, T1-5, and T1-7. M: marker; P: *proTcCHS-GUS* vector; WT EcoRI: control from nontransformed plants; 0: T0-X; 1: T1-1; 2: T1-5; 3: T1-7. (**e**) Histochemical staining and petiole slice of T0-X. (**f**) Reverse-transcription quantitative PCR analysis of *GUS* expression in T0-X, T0-1, T0-5, and T0-7. Bars indicate the mean ± SD. Bars with a different lower-case letter are significantly different (LSD test, *p* < 0.05). (**g**) Histochemical staining of nontransformed plants. (**h**) Histochemical staining of kanamycin-resistant seedlings. (**i**) Enlarged view of stained leaf. GT: capitate glandular trichomes; NGT: T-shaped nonglandular trichomes; LS: leaf stomata.

Of 300 explants, one resistant explant was obtained, namely T0-X, from which three lines, T0-1, T0-5, and T0-7, were achieved. Seeds of T0-1, T0-5, and T0-7 were harvested to obtain T1-1, T1-5, and T1-7, respectively. PCR, RT-qPCR, Southern blotting, and histochemical staining were used to further confirm the presence of T-DNA (Figure 3a) in transgenic *proTcCHS-GUS* plants. DNA and RNA extracted from leaf samples collected from these plants were used for analysis. The DNA from T0-X, T0-1, T0-5, and T0-7 produced positive bands that corresponded to the size of the *proTcCHS-GUS* fragment (362 bp), whereas the *VirG* gene fragment (383 bp) was not detected in T0-X, T0-1, T0-5 and T0-7 (Figure 3b,c). RT-qPCR analyses revealed the expression of *GUS* in T0-X, T0-1, T0-5, and T0-7; however, the expression level of *GUS* was much higher in T0-X and T0-1 than in T0-5 and T0-7 (Figure 3f). The results suggest that T0-5 and T0-7 may be transgenic chimeric plants. This is consistent with the results of PCR; that is, the PCR products of T0-5 and T0-7 were significantly less than T0-1 (Figure 3c). To confirm the transmission of T-DNA from the T_0_ plant to the T_1_ progeny, Southern blot analysis was performed on T0-X, T1-1, T1-5, and T1-7 plants. Multiple transgene insertions were observed in T0-X, but only a single transgene insertion was observed in T1-1, T1-5, and T1-7. In addition, the transgene insertions in T1-1, T1-5, and T1-7 differed (Figure 3d). To further confirm T-DNA insertion, T0-X and kanamycin-resistant seedlings (T1) were used for histochemical staining. Strong staining signals were observed in the leaf veins of the transformants (Figure 3e,h,i), but not in those of nontransformed plants (Figure 3g).

### 2.4. Verification of Transgenic proRbcS-TcCHS Plants

**Figure 4:**
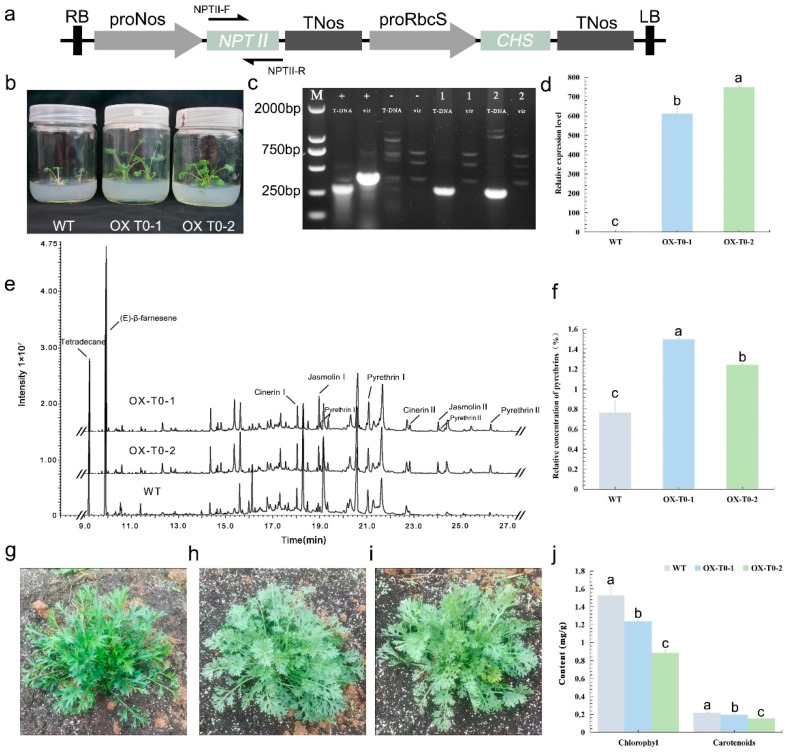
Analysis of transgenic *proRbcS-TcCHS* pyrethrum plants. (**a**) Schematic structure of *proRbcS-TcCHS* vector and primer locations (arrows). (**b**) OX T0-1 and OX T0-2 seedlings were screened on Murashige and Skoog medium supplemented with 30 mg L^−1^ kanamycin. WT: nontransformed plants. (**c**) Agarose gel image of PCR products showing amplification of the *NPTII* gene and *VirG* gene from genomic DNA of OX T0-1 and OX T0-2. M: marker; +: EHA105 harboring *proRbcS-TcCHS* vector; −: control from nontransformed plants; 1: OX T0-1; 2: OX T0-2; T-DNA: NPTII-F and NPTII-R were used for PCR; *vir*: VirG-F and VirG-R were used for PCR. (**d**) Reverse-transcription quantitative PCR analysis of *TcCHS* expression in OX T0-1 and OX T0-2. (**e**) GCMS chromatograms of OX T0-1, OX T0-2, and nontransformed plants. (**f**) Pyrethrin content in OX T0-1, OX T0-2, and nontransformed plants. (**g**) Nontransformed plant. (**h**) OX T0-1. (**i**) OX T0-2. (**j**) Chlorophyll and carotenoid contents of OX T0-1, OX T0-2, and nontransformed plants. Bars indicate the mean ± SD. Bars with different lower-case letters within a treatment differ significantly (*p* < 0.05).

*A. tumefaciens* (EHA105) harboring the *proRbcS-TcCHS* vector was used in the transformations (Figure 4a). The kanamycin-resistant plants OX T0-1 and OX T0-2 were used in PCR analysis (Figure 4b). The DNA from these plants produced positive bands that corresponded to the size of the *NPTII* gene fragment (273 bp), whereas the *VirG* gene fragment (383 bp) was not detected (Figure 4c). RT-qPCR analyses revealed expressions of *TcCHS* in both nontransformed and transgenic plants; however, the expression level of *TcCHS* was much higher in OX T0-1 and OX T0-2 than in nontransformed plants (Figure 4d). Next, the OX T0-1, OX T0-2, and nontransformed plants were subjected to gas chromatography–mass spectrometry (GCMS) analysis to detect differences in pyrethrin content (Figure 4e). The relative concentrations of pyrethrins in OX T0-1 (1.50%) and OX T0-2 (1.24%) were significantly higher than those in nontransformed plants (0.76%) (Figure 4f). The mass spectrum of identified peaks in GCMS analysis are shown in Table S1. The leaves of nontransformed plants (Figure 4g), OX T0-1 (Figure 4h), and OX T0-2 (Figure 4i) were used for the determination of chlorophyll and carotenoid contents. The contents of chlorophyll and carotenoids were decreased in OX T0-1 and OX T0-2 compared with those of nontransformed plants (Figure 4j). These results were consistent with the overall plant appearance; the leaves of OX T0-1 and OX T0-2 were more yellow than those of nontransformed plants.

## 3. Discussion

In recent years, remarkable progress in understanding pyrethrin biosynthesis has been made. The genes coding for enzymes involved in pyrethrin biosynthesis, such as *TcCDS* (subsequently renamed *TcCHS*, GenBank accession no. JX913537), have been cloned from pyrethrum [23]. The enzyme *TcCHS* functions at the first branch point in the pyrethrin biosynthesis pathway and catalyzes the conversion of dimethylallyl diphosphate to chrysanthemol [24]. Therefore, *TcCHS* plays an important role in pyrethrin biosynthesis. In the present study, *TcCHS* was overexpressed in pyrethrum, and the total pyrethrin content of transgenic plants was significantly higher than that in nontransformed plants. However, the stable overexpression of *TcCHS* significantly reduced the content of chlorophyll and carotenoids in pyrethrum. These results are similar to the previously reported stable overexpression of *CDS_CCI2* (an additional CHS; GenBank accession no. HQ235057) in tomatoes [25].

One damaged apical meristem can form many shoots [12]. In the current study, several transgenic shoots were obtained from one explant. Southern blot analysis revealed that, in the transgenic progenies of the transformants, a single copy of the transgene was integrated at different genomic locations (the pyrethrum genome is estimated to be approximately 7.1 Gb [26]). The leaves are rich in polysaccharides [27], which greatly hinders the use of Southern blotting. In the present study, digestion with *Hind*III and *Bam*HI failed; hence, the results are not discussed. In comparison with the negative control, false positive bands were excluded. Most transformants carried only one T-DNA insert in *A. tumefaciens*-mediated transformation [28]. However, single-copy integration events are uncommon in particle bombardment transformation [29]. Therefore, transgenic plants with single-copy integration events were obtained more readily by Agrobacterium-mediated transformation using shoot apical meristems as the target material.

The most important factor in the successful use of shoot apical meristems for genetic transformation is a deep and broad wounding procedure that exposes the shoot apical meristem to *A. tumefaciens* [12]. The present results are consistent with those of previous reports, and almost all kanamycin-resistant buds originated from explants with damaged (half) apical meristems. Seedling developmental stage also affected transformation efficiency. Shoots develop from shoot apical meristems, which comprise many cells [30], and during development the number of cells increases [31], making contact between cells and *A. tumefaciens* increasingly difficult. The preculture of explants before inoculation with *A. tumefaciens* leads to an increase in the frequency of genetic transformation [32]. The increase in success is likely because cell division events at the wound site are a requirement for T-DNA integration into cells [33]. However, the shoot tip of a seedling has many meristematic cells [12], and preculture is unnecessary in most cases [20,34,35]. In the present study, the preculture of pyrethrum explants with *A. tumefaciens* led to a decrease in transformation efficiency. The decrease might be because the capacity for cell division in the apical meristem of pyrethrum was greater than that in the newly formed callus. The application of cytokinin can promote the growth of lateral buds [36]. Given that a procedure using shoot apical meristems does not involve regeneration, optimal conditions can be obtained by optimizing only the concentration of cytokinin, without auxin [34,37]. Kanamycin is commonly used to screen transgenic plants and, in genetic transformation procedures based on organogenesis, the critical concentration of kanamycin often depends on its inhibition of regeneration [10]. However, this method is uncommon in genetic transformation with an apical meristem as the explant. Generally, necrosis is used to determine whether the shoot is kanamycin-resistant [19,21,35]. In the present study, non-necrotic shoots at the critical concentration of kanamycin were putative transgenic shoots. However, when kanamycin-resistant buds were stained with X-gluc, most kanamycin-resistant shoots were transgenic mosaics (Supplementary Materials Figure S1). Further screening and identification are required to obtain transgenic lines with stable characteristics.

## 4. Materials and Methods

### 4.1. In Vitro Seed Germination to Generate Seedling Shoot Apical Meristems

Mature seeds of pyrethrum (*T. cinerariifolium*) were collected in Yunnan Province (24°34′ N, 103°45′ E; 1780 m a.s.l.), China. Seeds were rinsed overnight in running water and then were surface-disinfected with 70% ethanol for 1 min, followed by three washes with sterilized distilled water, and sterilization with 10% H_2_O_2_ solution for 40 min, followed by five washes with sterilized distilled water. Seeds were germinated on Murashige and Skoog (MS) medium. Shoot apical meristems were sliced longitudinally into two halves and used as a source of material. Unless otherwise specified, all materials were incubated in a tissue culture room at 25 ± 2 °C under a 16 h:8 h (light:dark) photoperiod, with 3000 lx light provided by cool-white fluorescent lamps.

### 4.2. Optimization of Shoot Induction Conditions and Kanamycin Sensitivity of Explants

To establish an efficient shoot clump induction system in pyrethrum, explants were placed on MS medium supplemented with different concentrations of 6-BA (1.0, 1.5, 2.0, 2.5, or 3.0 mg L^−1^) in combination with 0.03 mg L^−1^ naphthaleneacetic acid (NAA). Cultures were incubated in the tissue culture room. After cultivation for 2 weeks, the frequency of explants that formed shoots was recorded. Each replicate was inoculated with 20 explants per Petri dish. All the subsequent data were collected from four biological replicates. The optimal medium identified in this experiment was used in subsequent Agrobacterium-mediated transformation experiments.

To determine the effective dose to select putative transformants, noninfected explants were cultured on shoot-induction medium supplemented with different concentrations of kanamycin (0, 10, 20, 30, 40, or 50 mg L^−1^). After 2 weeks of culture, the number of necrotic explants was counted. Each replicate was inoculated with 20 explants per Petri dish. All subsequent data were collected from four biological replicates. This experiment determined the critical kanamycin concentration for the subsequent Agrobacterium-mediated transformation experiments.

### 4.3. Optimization of Parameters Affecting Agrobacterium-Mediated Transformation

The following factors that could potentially affect Agrobacterium-mediated transformation were evaluated: explant status, preculture period, cocultivation period, and *A. tumefaciens* concentration. Nine different explant states representing nine possible combinations of seedling developmental stage (no true-leaf period, only true-leaf period, and true-leaf with cleft period) and apical meristem treatment (complete apical meristem, half apical meristem, and no apical meristem) were evaluated (Supplementary Materials Figure S2). To determine the optimal preculture duration, explants were precultured for 0, 1, 2, 3, or 4 d. Inoculated explants were cocultivated with one of nine possible combinations of period (1, 2, or 3 d) and concentrations of *A. tumefaciens* (OD_600_ = 0.3, 0.5, or 0.7). *A. tumefaciens* harboring the pBI121 vector (*pro35S-GUS*) were used in transformations. Infected explants were cultured on selection medium (see Table S2) for 14 d. The influence of the factors on transformation efficiency was evaluated by monitoring the frequency of explants that formed kanamycin-resistant shoots after selective culture. Experiments were performed using 20 explants with four replicates per treatment. Factors were optimized in the order they are listed in this section. When an optimal condition was established for a factor, that condition was maintained during the exploratory analyses of subsequent factors. Analyses of preculture and cocultivation periods were conducted on shoot-induction medium (see Table S2) in a tissue culture room at 25 ± 2 °C in the dark.

### 4.4. Vector and Preparation of Agrobacterium Suspension Cultures

The *proTcCHS-GUS* vector was constructed, in which the *TcCHS* promoter [38] was inserted into the binary vector pBI121 to drive the expression of *GUS*. The *proRbcS-TcCHS* vector was the same as that used in a previous study on chrysanthemum [39], and the vector was constructed in which the *TcCHS* (GenBank accession no. JX913537) gene was inserted into the binary vector pBINPLUS under the control of the promoter–terminator of chrysanthemum *rbcS1* [40]. In the *proRbcS-TcCHS* vector, *proRbcS* represents a strong constitutive rubisco small subunit promoter [41]. Next, the *proTcCHS-GUS* vector or *proRbcS-TcCHS* vector was introduced into the *A. tumefaciens* EHA105 strain. A single colony of *A. tumefaciens* strain EHA105 harboring the binary *proTcCHS-GUS* vector or the *proRbcS-TcCHS* vector was grown in 50 mL of liquid yeast extract broth supplemented with 50 mg L^−1^ kanamycin and 100 μM acetosyringone at 28 °C and shaken at 200 rpm. When the culture attained an OD_600_ of 0.6, bacterial cells were pelleted by centrifugation at 5000× *g* for 5 min. The pellet was resuspended in liquid MS medium supplemented with 100 μM acetosyringone. The *A. tumefaciens* suspension was used in transformation treatments.

### 4.5. Transformation and Selection of Putative Transgenic Plants

**Figure 5:**
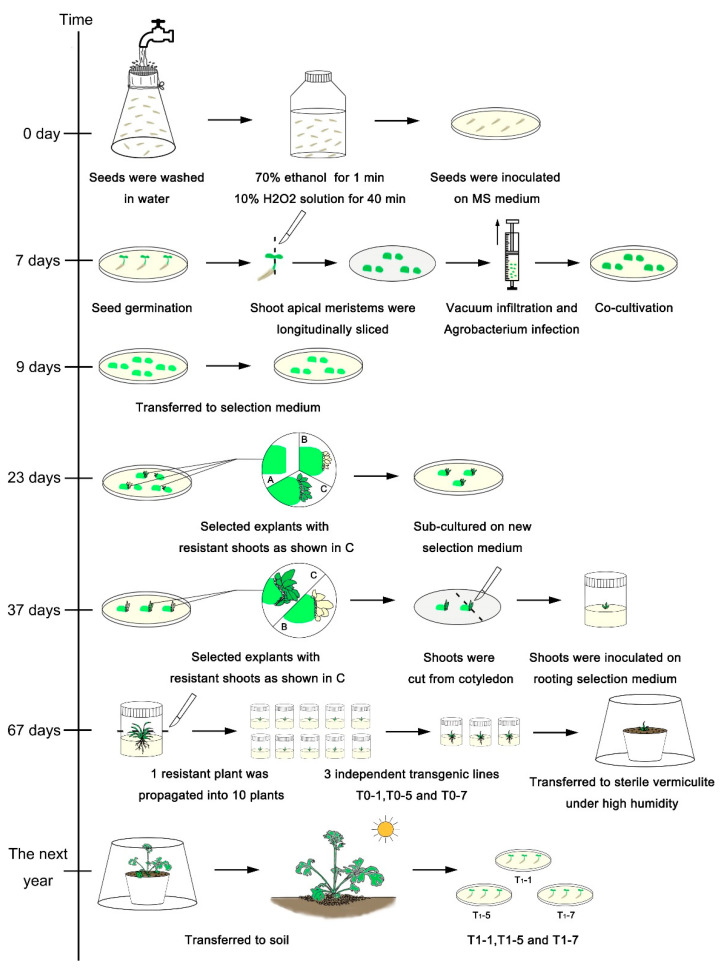
Workflow of pyrethrum transformation using shoot apical meristems of germinated seedlings. A: No regeneration. B: Blbino buds. C: Kanamycin-resistant buds.

The optimal parameters were used in subsequent Agrobacterium-mediated transformations to generate transgenic plants. *A. tumefaciens* harboring the *proTcCHS-GUS* vector or *proRbcS-TcCHS* vector were used in the transformations. Vacuum infiltration was used for *A. tumefaciens* infection, as described previously [42]. Explants in the no true-leaf period with half apical meristems that had been precultured for 0 d were transformed using *A. tumefaciens* strain EHA105 (OD_600_ = 0.5) and cocultivated for 2 d. After cocultivation, explants were transferred to the selection medium (see Table S2) and incubated in a tissue culture room. After cultivation for 2 weeks, explants with kanamycin-resistant shoots were subcultured on new selection medium (see Table S2). After 2 weeks of subculture, developing shoots were cut from the cotyledon and transferred to root-induction medium (see Table S2). Rooted shoots were transferred to sterile vermiculite (irrigated with MS solution) and maintained under high humidity for 30 d before transfer to soil. This process was used to obtain transgenic plants containing *proTcCHS-GUS* (T0-X, T0-1, T0-5, and T0-7) and those containing *proRbcS-TcCHS* (OX T0-1 and OX T0-2). The workflow for pyrethrum transformation is shown in Figure 5. Inverse PCR was used to identify the different transgenic lines [43]; T0-1, T0-5, and T0-7 were separated from the ramet population, as shown in the Supplementary Materials (Figure S3).

### 4.6. Analysis of proTcCHS-GUS in Transgenic Plants

To detect *proTcCHS-GUS* in the transgenic plants (T0-X, T0-1, T0-5, and T0-7), the forward specific primer proTcCHS-F (based on the promoter fragment of *TcCHS*, see Table S3) and the reverse specific primer GUS-R (based on a *GUS* fragment, see Table S3) were used in PCR analysis. A diagram showing the primer locations is shown in Figure 3a. The expected size of the PCR product was 362 bp. Furthermore, the PCR-based amplification of *VirG* genes (GenBank accession no. X62885.1) was used to test for *A. tumefaciens* contamination [44,45]; the forward specific primer VirG-F and the reverse specific primer VirG-R (based on the *VirG* gene fragment: see Table S3) were used, and the detection of *VirG* genes indicated *A. tumefaciens* contamination. DNA (50 ng) of putative transformed plants was analyzed using 2 × Taq Master Mix (Novoprotein, Nanjing, China). The PCR was conducted using the following conditions: 5 min at 94 °C, followed by 32 cycles of 30 s at 94 °C, 30 s at 58 °C, and 45 s at 72 °C, and a final extension of 5 min at 72 °C. It should be noted that DNA-free reagents and consumables are needed to eliminate false positives caused by DNA pollution in the environment.

Seeds from transgenic *proTcCHS-GUS* plants (T0-1, T0-5, and T0-7) were germinated aseptically and separately on MS medium. After 2 weeks, seeds were subcultured on MS medium supplemented with 30 mg L^−1^ kanamycin to screen transgenic progeny (T1-1, T1-5, and T1-7). To further confirm the transformation of T0-X, T1-1, T1-5, and T1-7 plants, Southern blot analysis was performed. DNA (30 µg) was digested with 50 U *Hind*III, *EcoRI,* or *Bam*HI with 4 µL of 10 × buffer in a total volume of 40 µL at 37 °C for 16 h. Digested DNA was separated in 0.8% agarose gel at 30 V for 16 h. The assay was performed using the DIG High Prime DNA Labeling and Detection Starter Kit II (Towin Biotechnology, Wuhan, China). The probe used was 21SB157NPT-II (Towin Biotechnology).

Next, RT-qPCR was used to analyze the expression level of *GUS* in transgenic plants (T0-X, T0-1, T0-5, and T0-7) and nontransformed plants grown on MS medium for 1 month. Relative quantitation by the real-time gene expression analysis of *GUS* and the reference gene *GAPDH* (GenBank accession no. ON398960) was performed using Applied Biosystems 7500 platform and SYBR^®^ green I with 6-carboxyl-X-rhodamine (Takara, Kusatsu, Japan), following a previously described protocol [46]. *GUS*-specific primers (GUS-RT-F, GUS-RT-R) and *TcGAPDH*-specific primers (TcGAPDH-RT-F, TcGAPDH-RT-R) were used (see Table S3). Total RNA was extracted using the Ultrapure RNA Kit (CWBIO, Beijing, China). The cDNA was synthesized using the Easy Script One-Step gDNA Removal and cDNA Synthesis Super Mix Kit (TransGen Biotech, Beijing, China) in accordance with the manufacturer’s instructions. Quantification of the transcript level was performed using a three-step program, which included (1) enzyme activation at 95 °C for 600 s, (2) 32 cycles of 95 °C for 10 s, 58 °C for 15 s, and 72 °C for 15 s, and (3) 95 °C for 15 s, from 65 °C to 97 °C for 60 s, for dissociation curve analysis. The expression of each gene was measured six times in total (three biological repeats with each comprising two technical repeats). PCR was used to determine that there was no DNA contamination in the RNA. The methods and results are shown in the Supplementary Materials (Figure S4).

In order to detect *proTcCHS-GUS* in the transgenic plants, histochemical staining was conducted with T0-X and the transgenic progeny T1-1, T1-5, and T1-7 using a *GUS* Staining Kit (Coolaber, Beijing, China). In theory, transgenic plants appear blue.

### 4.7. Analysis of proRbcS-TcCHS in Transgenic Plants

To detect *proRbcS-TcCHS* in the transgenic plants (OX T0-1 and OX T0-2), specific primers based on the *NPTII* gene fragment in T-DNA were used for the PCR [10]. The forward specific primer NPTII-F (see Table S3) and the reverse specific primer NPTII-R (see Table S3) were used. A diagram showing the primer locations can be seen in Figure 4a. The expected size of the PCR product was 273 bp. The primer VirG-F and VirG-R were used to eliminate false positives. The PCR reaction was conducted using the conditions described in Section 4.6.

RT-qPCR was used to analyze the expression level of *TcCHS* in transgenic plants (OX T0-1 and OX T0-2) and nontransformed plants grown on MS medium for 1 month. *TcCHS*-specific primers (TcCHS-RT-F, TcCHS-RT-R, see Table S3) were designed according to [47]. The experimental method is the same as outlined in Section 4.6.

### 4.8. Phenotypic Trait Analysis of OX T0-1 and OX T0-2

Gas chromatography–mass spectrometry was used to analyze the secondary metabolites of OX T0-1, OX T0-2, and nontransformed plants grown on MS medium for 2 months. Leaf samples (500 mg) were frozen in liquid nitrogen and ground using a mortar and pestle, then transferred to a 5 mL tube, to which 3 mL MTBE with 0.01 ng mL^−1^ tetradecane was added as an internal standard. The tube was vortexed for 3 min at maximum speed, and incubated at 24 °C with rotation at 50 rpm. The MTBE phase was then dried using Na_2_SO_4_. For GCMS analysis, a 1 µL aliquot of the extract was injected into the GC/MS-QP2010Ultra apparatus (Shimadzu Corporation, Kyoto, Japan) with an HP-5 MS column. The relative concentration of pyrethrins was calculated as the ratio of the area of detected compounds and the area of the internal standard [8]. Helium (1.4 mL min^−1^) was used as the carrier gas. The injection temperature was set at 240 °C. The oven temperature program was as follows: initial temperature 50 °C, followed by a ramp from 50 °C to 110 °C at the rate of 20 °C min^−1^, held for 1 min, and then from 110 °C to 240 °C at the rate of 10 °C min^−1^, held for 3 min, and finally increased to 300 °C at the rate of 5 °C min^−1^, and held for 3 min. Volatiles were identified by comparison of their retention time and mass fragmentation with those in the literature and the NIST library.

For chlorophyll and carotenoid analyses, large leaves (>25 mm) from field-grown transgenic and nontransformed plants were used. The large leaves of field-grown plants were selected as described previously [48]. Leaf samples (100 mg) were ground in liquid nitrogen and extracted with 5 mL of 95% ethanol for 48 h in the dark at 20 °C. The concentrations were measured as described previously [49].

### 4.9. Statistical Analyses

All experimental data are expressed as the mean ± standard error (SE). Statistical differences between means were assessed using the least significant difference (LSD) test. Percentage data were arcsin square root transformed before performing an analysis of variance with SPSS v17.0 software (SPSS, Chicago, IL, USA). Differences at *p* < 0.05 were considered to be statistically significant.

## 5. Conclusions

In this study, meristematic tissues of germinated seedlings were used to develop an effective genetic transformation system in pyrethrum. Factors affecting transformation efficiency were optimized. Optimal conditions included explants at the “no true leaf” stage with a half apical meristem, an *A. tumefaciens* cell density of OD_600_ = 0.5, cocultivation for 2 d, and the incorporation of 1.5 mg L^−1^ 6-BA and 30 mg L^−1^ kanamycin into the selection medium. Under the optimized conditions, the average transformation efficiency was 0.83%. The procedure was used to separately transform *proTcCHS-GUS* and *proRbcS-TcCHS* vectors into pyrethrum. The presence of *proTcCHS-GUS* in T1-1, T1-5, and T1-7 was verified using histochemical staining and Southern blotting. T1-1, T1-5, and T1-7 exhibited single-copy integration of the transgene into the genome at different positions, and therefore represented distinct transgenic lines. The presence of *proRbcS-TcCHS* in OXT0-1 and OXT0-2 was confirmed by PCR and RT-qPCR. The content of pyrethrins in transgenic plants was significantly higher than that in nontransformed plants. Thus, in pyrethrum—an important industrial crop plant previously recalcitrant to regeneration during transformation—Agrobacterium-mediated transformation using shoot apical meristem explants provides a sound foundation for molecular breeding and functional genomic research.

## Data Availability

Data sets generated and analyzed in the current study are available from the corresponding author on reasonable request.

## Supplementary Materials

The following supporting information can be downloaded at: https://www.mdpi.com/article/10.3390/plants11121575/s1. Figure S1: Resistant shoots are transgenic mosaics with a uniform expression. Figure S2: Nine different statuses of pyrethrum explants. Figure S3: Verification of the *proTcCHS-GUS* transgenic lines T0-1, T0-5, and T0-7. Figure S4: Detection of genomic DNA contamination in RNA. Table S1: Mass spectrum of identified peaks in the pyrethrum detected by GC-MS. Table S2: Different media and composition. Table S3: Primers used in the experiment. Table S4: gDNA removal and cDNA synthesis without reverse transcriptase. Table S5: gDNA removal and cDNA synthesis with reverse transcriptase.
