# Supplementary material for: Overexpression of TcCHS Increases Pyrethrin Content When Using a Genotype-Independent Transformation System in Pyrethrum (Tanacetum cinerariifolium)
Source: Plants (Basel). 2022 Jun 15;11(12):1575. doi: 10.3390/plants11121575 (PMC9229838; doi:10.3390/plants11121575)
Supplement: Supplementary file 1 [file plants-11-01575-s001.zip › plants-1696797-supplementary.pdf]

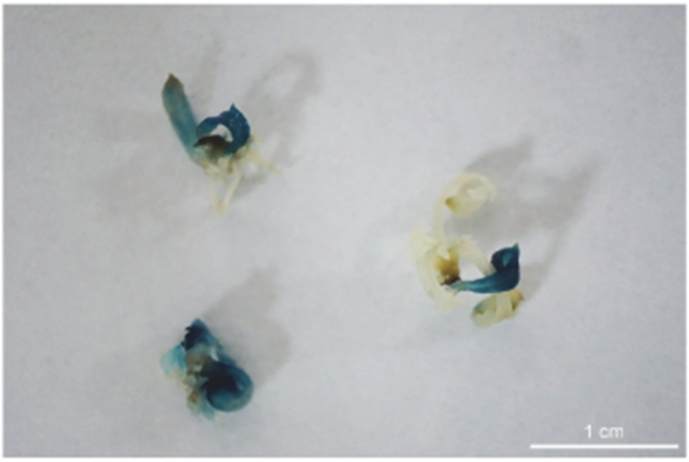

Figure S1. Resistant shoots are transgenic mosaics with a uniform expression.

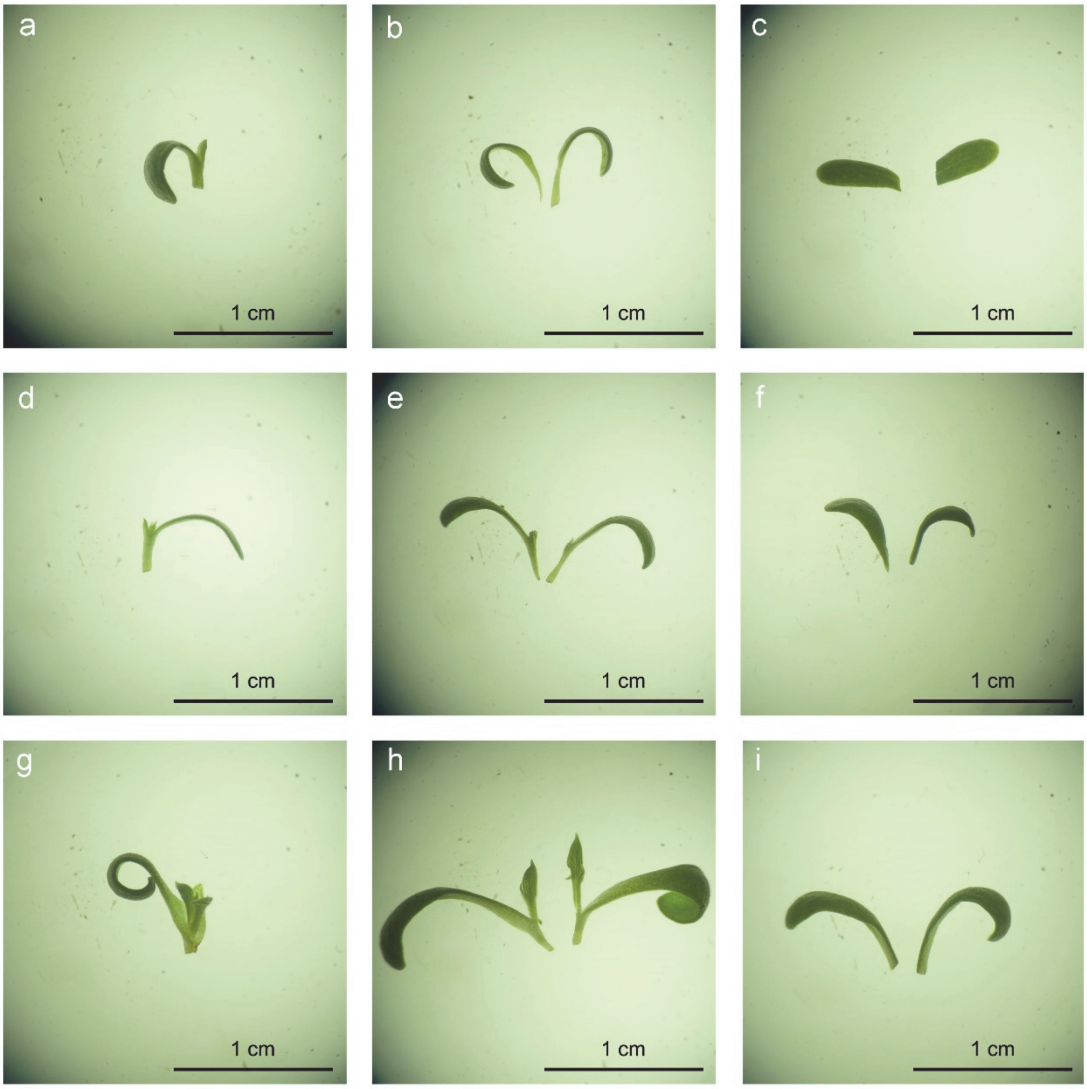

Figure S2. Nine different statuses of pyrethrum explants. (**a**) No true leaf period and complete apical meristem. (**b**) No true leaf period and half apical meristem. (**c**) No true leaf period and no apical meristem. (**d**) Just reached true leaf period and complete apical meristem. (**e**) Just reached true leaf period and half apical meristem. (**f**) Just reached true leaf period and no apical meristem. (**g**) True leaf

with cleft period and complete apical meristem. (**h**) True leaf with cleft period and half apical meristem. (**i**) True leaf with cleft period and no apical meristem. Bar = 1.0 cm.

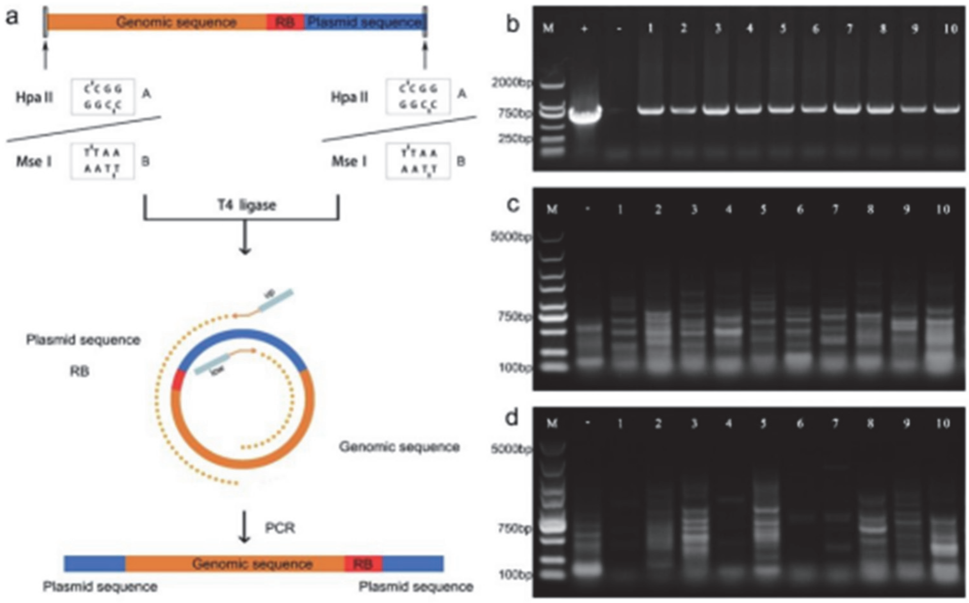

Figure S3. Verification of the *proTcCHS-GUS* transgenic lines T0-1, T0-5, and T0-7. (**a**) A schematic of the reverse polymerase chain reaction (PCR) used in this study. (**b**) PCR-based detection of NPT II in the ramet population. (**c**) Results of an inverse PCR using HpaII. (**d**) Results of an inverse PCR using MseI. M: marker. +: control from *proTcCHS-GUS* plasmid. −: control from wild type plants. 1 to 10: T0-1 to T0-10.

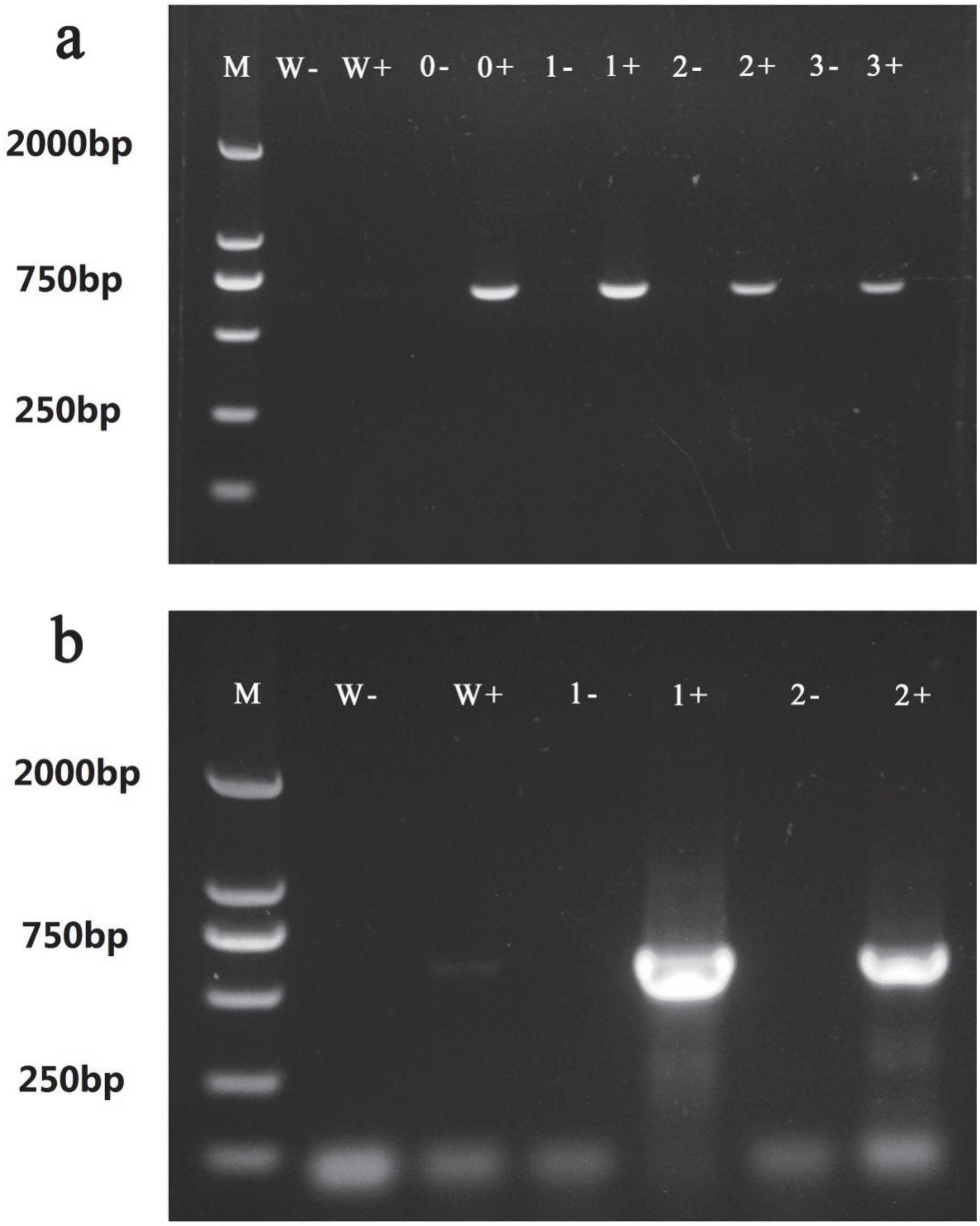

Figure S4. Detection of genomic DNA contamination in RNA. (**a**) GUS-RT-F and GUS-652-R were used for T0-X, T0-1, T0-5, and T0-7. M: marker. W: Wild plant. 0: T0-X. 1: T0-1. 2: T0-5. 3: T0-7. (**b**) TcCHS-RT-F and TcCHS-end-R were used for OX T0-1 and OX T0-2. M: marker. W: Wild plant. 1: OX T0-1. 2: OX T0-2. −: RNA were treated according to Table S4. +: RNA were treated according to Table S5.

Table S1. Mass spectrum of identified peaks in the pyrethrum detected by GC-MS.

RT(min)	Mass Spechmn Compound	Mass Spechwn NIST
7.08	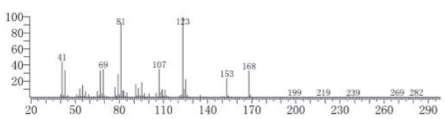	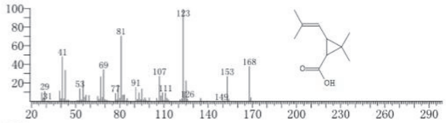
9.84	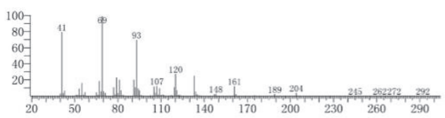	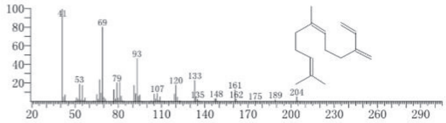
15.93	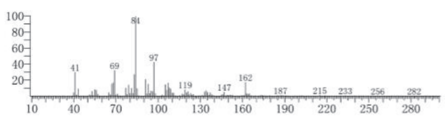	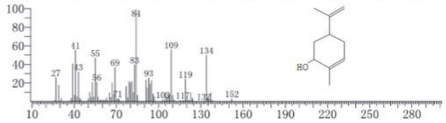
17.97	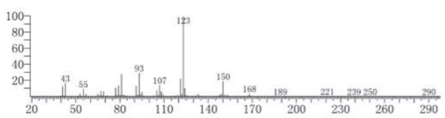	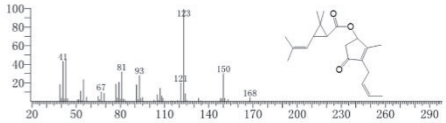
18.89	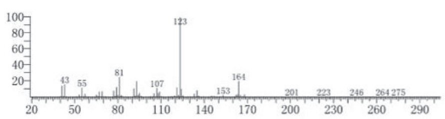	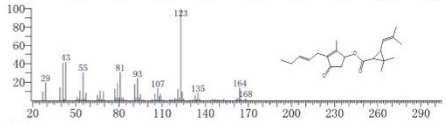
18.96	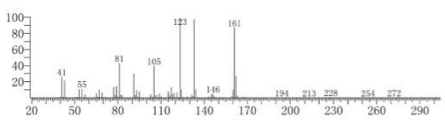	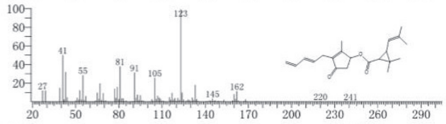
22.76	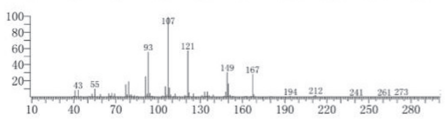	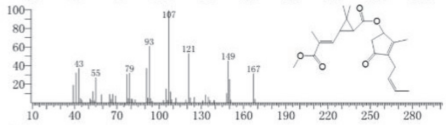
23.96	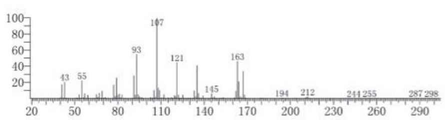	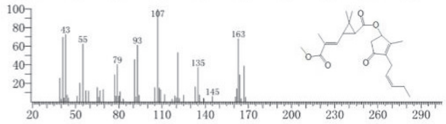
24.03	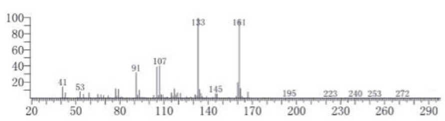	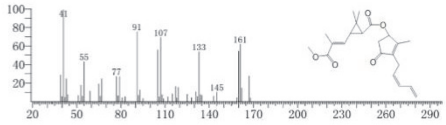

Table S2. Different media and composition.

Name of Media	Composition of Media
Shoot-induction medium	4.42 g/L Murashige and Skoog, 3% (*w*/*v*) sucrose, 1.5 mg/L 6-BA, 0.03 mg/L NAA, 0.75% (*w*/*v*) agar, pH = 5.8 (NaOH)
Selection medium	4.42 g/L Murashige and Skoog, 3% (*w*/*v*) sucrose, 1.5 mg/L 6-BA, 0.03 mg/L NAA, 0.75% (*w*/*v*) agar, 30 mg/L Kanamycin, 400 mg/L Cefotaxime, pH = 5.8 (NaOH)
Root-induction medium	4.42 g/L Murashige and Skoog, 3% (*w*/*v*) sucrose, 0.75% (*w*/*v*) agar, 30 mg/L Kanamycin, 400 mg/L Cefotaxime, pH = 5.8 (NaOH)

Table S3. Primers used in the experiment.

Primers	Sequence (5′-3′)	Note
proTcCHS-F	GCTATTATAAAATCCCGTGTCTATG	PCR
GUS-R	TGGCCTGCCCAACCTTTCG	PCR
NPTII-F	GGCTATGACTGGGCACAACAGACAA	PCR
NPTII-R	CTCGGCAGGAGCAAGGTGAGATGAC	PCR
Inverse PCR-F	TCCTGTCAAACACTGATAG	Inverse PCR
Inverse PCR-R	AGGATATATTGGCGGGTAAACC	Inverse PCR
GUS-RT-F	AACCGTTCTACTTTACTGGCTTTGG	qRT-PCR
GUS-RT-R	GCATCTCTTCAGCGTAAGGGTAAT	qRT-PCR
TcCHS-RT-F	CATCTTCTGGACCTCTTCAATGAG	qRT-PCR
TcCHS-RT-R	GTACTGAACAATCCGACGGTTAAG	qRT-PCR
TcGAPDH-RT-F	AAGGAGGAATCTGAAGGAAAGCTG	qRT-PCR
TcGAPDH-RT-R	GTTGTTGTTCAAAGCGATTCCAGC	qRT-PCR
VirG-F	GCCGACAGCACCCAGTTCAC	PCR
VirG-R	CCTGCCGTAAGTTTCACCTCACC	PCR
NPTII-orf-F	ATGATTGAACAAGATGGATTGCACGC	PCR
NPTII-orf-R	TCAGAAGAACTCGTCAAGAAGGCG	PCR
TcCHS-end-R	TGTCCCTTATACATCTTTTCCAGAC	PCR
GUS-652-R	GTGATGATAATCGGCTGATGCAG	PCR

Table S4. gDNA removal and cDNA synthesis without reverse transcriptase.

Component	Addition Amount
Oligo (dT) 18 Primer	1 μL
2 × ES Reaction Mix	10 μL
EasyScript RT/RI Enzyme Mix	0 μL
gDNA Remover	1 μL
RNase-Free ddH_2O	6 μL
RNA (500 ng/μL)	2 μL

Table S5. gDNA removal and cDNA synthesis with reverse transcriptase.

Component	Addition Amount
Oligo (dT) 18 Primer	1 μL
2 × ES Reaction Mix	10 μL
EasyScript RT/RI Enzyme Mix	1 μL
gDNA Remover	1 μL
RNase-Free ddH_2O	5 μL
RNA (500 ng/μL)	2 μL
